# An RNA replicon system to investigate promising inhibitors of feline coronavirus

**DOI:** 10.1128/jvi.01216-23

**Published:** 2024-01-18

**Authors:** Kimberly Schmied, Rosina Ehmann, Claudia Kristen-Burmann, Nadine Ebert, Güliz Tuba Barut, Lea Almeida, Jenna N. Kelly, Lisa Thomann, Hanspeter Stalder, Reto Lang, Gergely Tekes, Volker Thiel

**Affiliations:** 1 Institute of Virology and Immunology (IVI), Bern and Mittelhäusern, Switzerland; 2 Department of Infectious Diseases and Pathobiology, Vetsuisse Faculty, University of Bern, Bern, Switzerland; 3 Institute of Virology, Justus Liebig University Giessen, Giessen, Germany; 4 Multidisciplinary Center for Infectious Diseases (MCID), University of Bern, Bern, Switzerland; 5 European Virus Bioinformatics Center (EVBC), Jena, Germany; 6 Graduate School for Cellular and Biomedical Sciences, University of Bern, Bern, Switzerland; Loyola University Chicago - Health Sciences Campus, Maywood, Illinois, USA

**Keywords:** feline infectious peritonitis, feline coronavirus, replicon, antiviral agents

## Abstract

Feline infectious peritonitis (FIP) is a fatal feline disease, caused by a feline coronavirus (FCoV), namely feline infectious peritonitis virus (FIPV). We produced a baby hamster kidney 21 (BHK) cell line expressing a serotype I FCoV replicon RNA with a green fluorescent protein (GFP) reporter gene (BHK-F-Rep) and used it as an *in vitro* screening system to test different antiviral compounds. Two inhibitors of the FCoV main protease (M^pro^), namely GC376 and Nirmatrelvir, as well as the nucleoside analog Remdesivir proved to be effective in inhibiting the replicon system. Different combinations of these compounds also proved to be potent inhibitors, having an additive effect when combined. Remdesivir, GC376, and Nirmatrelvir all have a 50% cytotoxic concentration (CC50) more than 200 times higher than their half-maximal inhibitory concentrations (IC50), making them important candidates for future *in vivo* studies as well as clinically implemented drug candidates. In addition, results were acquired with a virus infection system, where *Felis catus* whole fetus 4 (Fcwf-4) cells were infected with a previously described recombinant GFP-expressing FIPV (based on the laboratory-adapted serotype I FIPV strain Black) and treated with the most promising compounds. Results acquired with the replicon system were comparable to the results acquired with the virus infection system, demonstrating that we successfully implemented the FCoV replicon system for antiviral screening. We expect that this system will greatly facilitate future screens for anti-FIPV compounds and provide a non-infectious system to study and evaluate drug-resistant mutations that may emerge in the FIPV genome.

**IMPORTANCE:**

FIPV is of great significance in the cat population around the world, causing 0.3%–1.4% of feline deaths in veterinary practices (2). As there are neither effective preventive measures nor approved treatment options available, there is an urgent need to identify antiviral drugs against FIPV. Our FCoV replicon system provides a valuable tool for drug discovery *in vitro*. Due to the lack of cell culture systems for serotype I FCoVs (the serotype most prevalent in the feline population) (2), a different system is needed to study these viruses. A viral replicon system is a valuable tool for studying FCoVs. Overall, our results demonstrate the utility of the serotype I feline coronavirus replicon system for antiviral screening as well as to study this virus in general. We propose several compounds representing promising candidates for future clinical trials and ultimately with the potential to save cats suffering from FIP.

## INTRODUCTION

Feline infectious peritonitis (FIP) is a viral disease, globally affecting wild and domestic felines (1, 2). It is almost invariably a deadly disease without any current legal options for a cure (3) nor an effective vaccine (4) in most parts of the world. The causative agent, the feline infectious peritonitis virus (FIPV), is a positive-sense single-stranded RNA virus belonging to the family *Coronaviridae*, species of *Alphacoronavirus 1* and subspecies *feline coronavirus* (FCoV). It is closely related to not only coronaviruses infecting animals, like the canine coronavirus (CCoV) infecting dogs, or the transmissible gastroenteritis virus (TGEV) infecting pigs but also to human coronaviruses like human coronavirus 229E (HCoV-229E) (5). Belonging to the same subspecies of feline coronavirus is the feline enteric coronavirus (FECV), which is widely spread in the European cat population, affecting up to 90% of cats in multi-cat households (6). FECV replicates in the intestinal epithelium and accordingly sheds in the feces but causes no symptoms or only mild enteritis in infected cats (7–10). However, in about 5%–12% of the FECV-infected individuals, the lethal FIPV can emerge within infected cats (4, 6). Different mutations in the FECV genome, which currently are not fully understood, can lead to the pathotype switch from FECV to FIPV displaying a changed tropism toward monocytes, spreading systemically within the infected cat and losing the ability to efficiently replicate in the intestine. Consequently, FIPV is not able to efficiently shed *via* feces (11).

While FIPV is not readily transmitted to other cats, the systemic spread of FIPV within individual cats causes the life-threatening disease FIP. As no legal treatment options are currently available in Europe as well as most parts of the world (3), there is a great need to find and implement antiviral compounds. There are several reports of successful treatment of FIP in experimental settings or clinical trials using inhibitors that target different steps in the coronavirus life cycle, such as virus entry, polyprotein processing, and replication (12–20). Since the emergence of SARS-CoV-2, the search for antiviral compounds has intensified. Particularly, antivirals targeting conserved coronavirus enzymes, such as viral proteases or RNA-dependent RNA polymerase, appear to be promising and could be explored for FIP treatment. Amodiaquine is a compound that has an inhibitory effect not only on entry but also suspected to influence later time points of the virus life cycle. It belongs to the 4-Aminoquinoline family and was initially developed for Malaria treatment (21). It is suspected that Amodiaquine has an inhibitory effect on different viruses by increasing endosomal pH (22) and is also effective in different flavivirus replicon-expressing cell lines (23). Multiple studies have shown it can also enter hard-to-reach tissues (e.g., the nervous system) which are not accessible for many other compounds (24). Cook et al. showed, in a recently published screen of antivirals, that the combination of Amodiaquine with GC376, an inhibitor of the coronavirus main protease M^pro^, seemed to be most efficacious against feline coronaviruses (25). The mode of action of GC376 is *via* covalently bonding with a catalytic cysteine residue of the M^pro^ (12) and shows promising inhibitory effects against FIPV *in vitro* and *in vivo* (18–20). With the same mode of action as GC376, Nirmatrelvir (PF-07321332) is an effective inhibitor of SARS-CoV-2 and has also been licensed for the treatment of Covid-19 patients as a coformulation with Ritonavir (Paxlovid) (26–28). However, neither its efficacy against feline coronaviruses nor *in vivo* therapeutic safety in felines has been tested yet. Remdesivir or its active form GS-441524 is an adenosine analog and inhibits the coronavirus RNA-dependent RNA polymerase.

More recently, an orally bioavailable Remdesivir Analog V2043 (29) has been developed for the treatment of SARS-CoV-2 infections. GS-441524 has been tested against FIPV *in vitro* as well as *in vivo* with experimentally infected cats, all of which showed very promising results both for efficacy and safety (13–17).

As most of the above-mentioned potential inhibitors have different modes of action, some combinations could be of interest, but so far there is very limited *in vitro* data and no *in vivo* studies on the use of combined antivirals against FIPV. Challenges during the treatment of FIP include addressing the dry or granulomatous form of FIP as well as reaching target tissues behind anatomical barriers like the brain in neurological forms of FIP (12). Combination therapy may be an efficient way of tackling these problems and limiting resistance development. A major bottleneck when systematically screening for or assessing anti-FIPV compounds (and combinations thereof) is the lack of robust cell culture systems to isolate and propagate FECV or FIPV. There are two serotypes known for FECV and FIPV but only a few virus isolates are available. Most of these isolates are from serotype II, which is much less prevalent than serotype I. These isolates were initially FIP viruses that have been adapted to cell culture and most are still difficult to propagate. This limitation has hampered research on FIP in general, particularly the systematic assessment of anti-FIPV compounds.

We show here the development of an FECV replicon cell line based on a field serotype I FECV generated by reverse genetics (recFECV) (30). Although recFECV could not be propagated in cell culture following rescue from cloned full-length DNA, replication of recFECV in cats has been achieved following a rescue procedure involving one passage in cell culture followed by experimental infection of cats (30). However, we show that a recFECV-derived replicon RNA, comprised of the 5′-untranslated region (5′-UTR), the replicase ORFs 1a and ORF1b, a selection gene (neomycin resistance gene), reporter gene (green fluorescent protein gene), the nucleocapsid gene, and the 3′-UTR, is replication competent in cell culture. Following electroporation of the FECV replicon RNA into Baby Hamster Kidney 21 (BHK) cells, we could select green-fluorescent cells stably expressing the replicon RNA (BHK-F-Rep cells). Since the BHK-F-Rep cells lack critical structural proteins needed for the formation of progeny virus particles, no infectious virus particles are formed allowing the study of replication processes of highly pathogenic viruses under BSL1 and BSL2 settings. We applied the BHK-F-Rep cells to evaluate antiviral compounds and demonstrate the replicon system is well suited as a screening platform for antiviral compounds interfering with the genome replication process, even for viruses that cannot be propagated in cell culture (31–35).

## RESULTS

### Generation of FECV replicon RNA-containing cells

**Fig 1:**
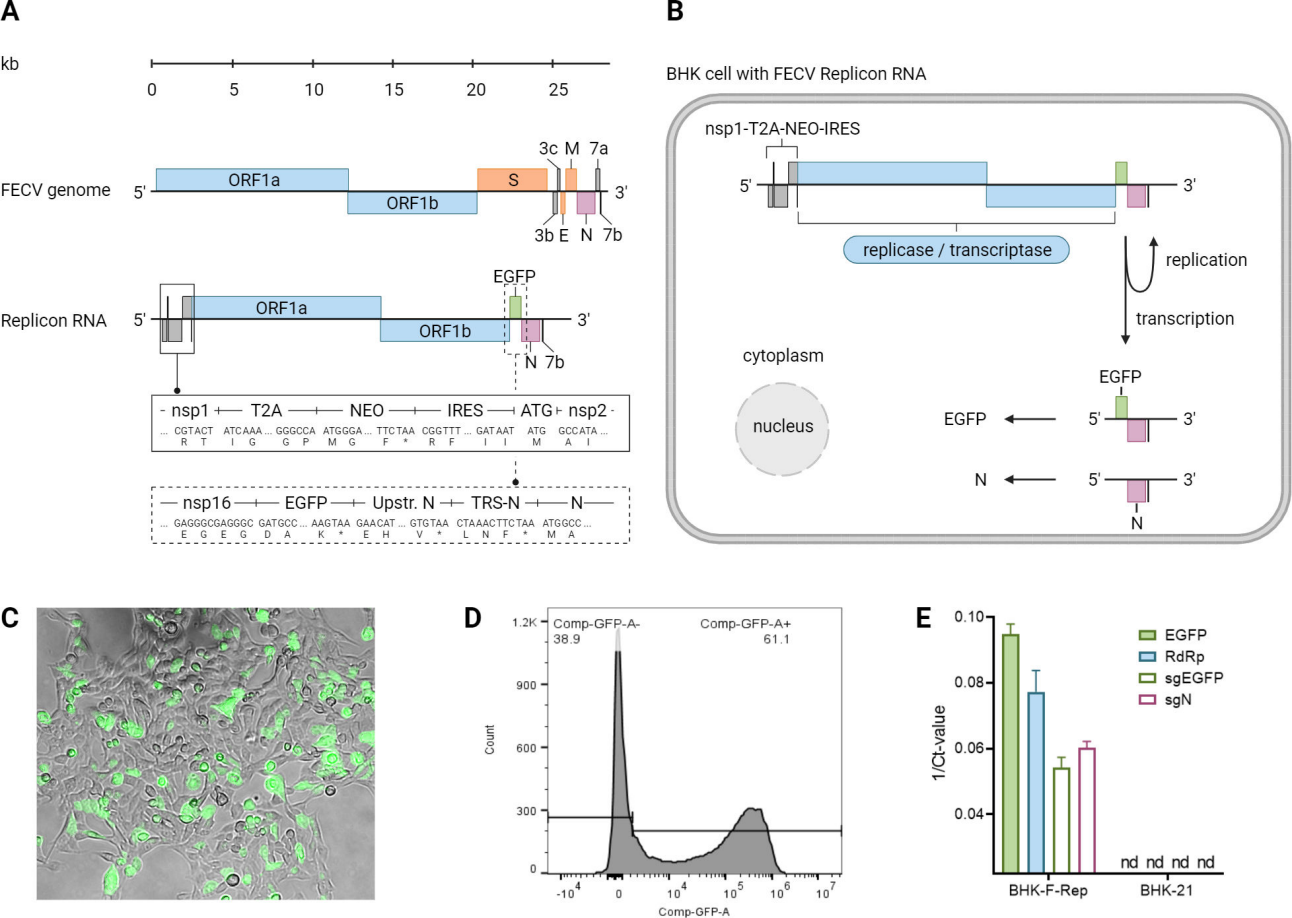
Illustration of the vrecFECVrep genome and function inside the cell including visualization and detection *via* flow cytometry. (**A**) vrecFECVrep genome compared to the FECV genome was illustrated from the 5′ UTR to the 3’UTR. The modifications introduced to the sequence upstream and downstream of ORF1ab are presented in solid and dashed rectangles, respectively. Kb = kilobases, ORF = open reading frame, S = spike, E = envelope, M = membrane, *N* = nucleocapsid, EGFP = enhanced green fluorescent protein, nsp = non-structural protein, T2A = TaV 2A-like element from Thosea asigna virus, NEO = neomycin selection gene, IRES = internal ribosome entry site, ATG = start codon, Upstr. *N* = part of the M protein upstream of N, TRS-N = transcription-regulating sequence of N. (**B**) Illustration of the BHK-F-Rep cell. The negative-sense vrecFECVrep RNA genome is replicated and transcribed into negative-sense full-length and subgenomic RNAs in the cytoplasm. The two subgenomic mRNAs encoding EGFP and N are then translated into proteins. (**C**) BHK-F-Rep cell colony expressing GFP was obtained after electroporation and selection. (**D**) Representation of the BHK-F-Rep cells in flow cytometry. (**E**) Expression of the RNA-dependent-RNA-Polymerase (RdRp), EGFP, subgenomic GFP (sgEGFP), and subgenomic nucleocapsid (sgN) gene as RT-qPCR results in BHK-F-Rep and BHK-21 cells. nd = not detected. Created with BioRender.com.

The previously described reverse-engineered serotype I FECV field isolate was used as the basis to construct an FECV replicon RNA (30). The minimal requirements for autonomous replication of a coronavirus RNA are the presence of 5′- and 3′-UTRs, the replicase gene (ORFs 1a and 1b), and the nucleocapsid gene (36). Comparing the full-length FECV sequence with the cloned DNA of the FECV replicon RNA shows the replicon does not contain the FECV structural genes, spike (S), membrane (M), and envelope (E), and the accessory genes ORF3a, 3b, 3c, and ORF 7a, 7b. To allow for the selection of cells containing an autonomously replicating FECV replicon RNA, we made use of a selection strategy that we have successfully implemented for a replicon RNA of the closely related human coronavirus 229E (HCoV-229E) (37). Downstream of the non-structural protein 1 (nsp1) coding sequence, we inserted the sequence of a 2A-like autoprocessing peptide of the *Thosea asigna* virus (TaV-2A) (38) followed by the gene encoding neomycin resistance that ends with a stop codon. To ensure translation of FECV nsps 2–16, we inserted an internal ribosomal entry site (IRES) from encephalomyocarditis virus (EMCV) downstream of the neomycin resistance gene and upstream of the nsp2 coding sequence. Finally, to monitor the replication of the FECV replicon RNA, we inserted the green fluorescent protein reporter gene downstream of FECV ORF1b that should be expressed *via* a subgenomic mRNA driven by the FECV transcription regulatory sequence (TRS) of the FECV spike gene. The overall FECV replicon RNA structure and its proposed intracellular replication and transcription are illustrated in Fig. 1A and B.

FECV replicon RNA was cloned using vaccinia virus as the cloning vector (see Materials and Methods), then a T7-RNA polymerase transcript resembling the FECV replicon RNA was electroporated into BHK cells that had received a plasmid DNA expressing the nucleocapsid protein one day before. After 48 hours of electroporation, cells were collected and seeded at different dilutions with the G418-containing medium to select for neomycin-resistant cells. As shown in Fig. 1C, individual cell colonies have been obtained that include green fluorescent cells, indicating successful recovery of BHK cells containing the FECV replicon RNA (BHK-F-Rep cells). After further propagation under G418 selection, stocks of BHK-F-Rep cells were obtained that could be stored frozen and displayed green fluorescence in 40%–70% of cells (Fig. 1D). Transcription of the FECV replicon RNA was confirmed to be as proposed *via* real-time RT-PCR including transcription of subgenomic RNA of both the EGFP Gene, as well as the N-Gene (Fig. 1E). Sequencing analysis of the replicon RNA of BHK-F-Rep cells confirmed the identity of the FECV replicon RNA and revealed adaptive mutations in nsp1, nsp12, nsp13, and nsp14, and interestingly also in the TaV-2A and IRES elements, suggesting that a balance between the expression of the replicase gene and cell survival has been established (Table S1).

### Assessment of antiviral compounds using BHK-F-Rep cells

**Fig 2:**
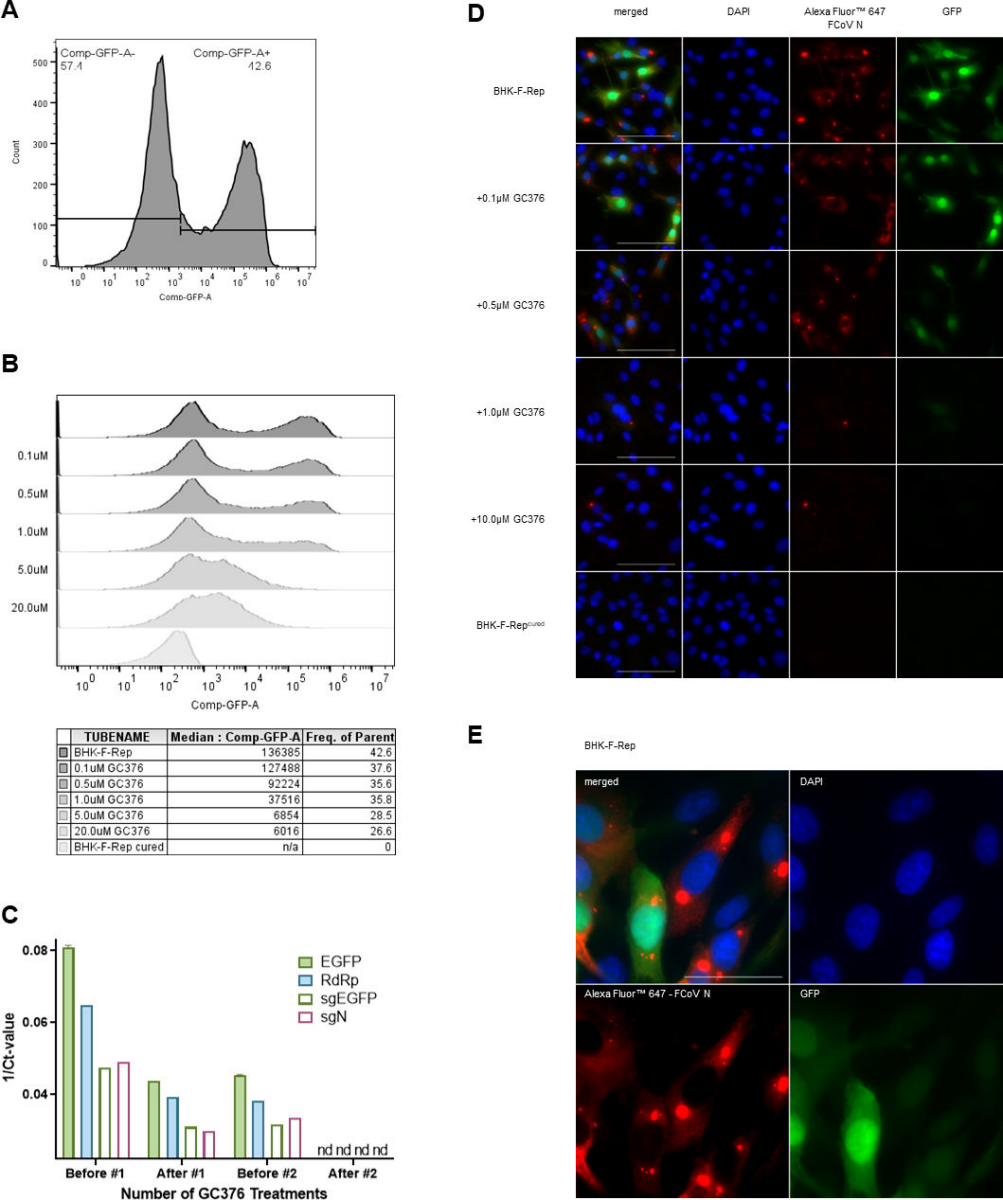
Representative flow cytometry analysis of BHK-F-Rep in response to inhibitors. The BHK-F-Rep were treated with the different compounds directly after seeding and kept at 37°C and 5% CO2 for 96 hours and subsequently analyzed with flow cytometry or immunofluorescence staining. (**A**) Untreated BHK-F-Rep with gating of GFP+ and GFP− cells. (**B**) An example of dose-response with different drug concentrations was analyzed with flow cytometry. The median GFP signal decreases with increasing GC376 concentration. (**C**) Establishment of the BHK-F-Rep^cured^ cell line with two treatments of the BHK-F-Rep cells with 20 µM of GC376. RdRp, EGFP, subgenomic EGFP (sgEGFP), and subgenomic N (sgN) expression before, during, and after the BHK-F-Rep cells were cured were analyzed with RT-qPCR. The BHK-F-Rep^cured^ cells were then used as a negative control for all experiments. nd = not detected. (**D**) An example of dose-response with different drug concentrations was analyzed with immunofluorescence to show the correlation between the GFP signal and the expression of the N-Protein. Blue = DAPI stain of the nucleus, green = GFP signal, red = Alexa Fluor 647 signal of the FCoV N-Protein staining, scale bar = 50 µm. (**E**) Close-up immunofluorescence image illustrating the different distribution of the expression of N-Protein versus GFP expression in the BHK-F-Rep cells. Blue = DAPI stain of the nucleus, green = GFP signal, red = Alexa Fluor 647 signal of the FCoV N-Protein staining, scale bar = 20 µm

We next addressed whether the BHK-F-Rep cells are suitable for assessing antiviral compounds. The BHK-F-Rep cells would specifically allow the evaluation of inhibitors that target viral replication, which includes basic processes such as RNA synthesis and viral polyprotein processing. We, therefore, used GC376 to establish a readout for inhibition based on the reporter protein GFP expression. GC376 is known to target the highly conserved coronavirus M^pro^ activity and its anti-FIPV activity has already been demonstrated *in vivo* (18, 20). BHK-F-Rep cells were freshly seeded (25,000 cells per 12-well dish) and grown for 96 hours in the culture medium without G418 but containing GC376 ranging from 0.1 to 20 micromolar (µM). GFP expression was assessed by flow cytometry. We observed a dose-dependent reduction of both GFP-positive cells and median fluorescent intensity (Fig. 2). Since we observed that the percentage of GFP positive in untreated BHK-F-Rep cells can vary between 40% and 70% cells, dependent on the stock used for the experiments, we decided to use the median fluorescent intensity of GFP-positive cells as a read-out in further experiments. Notably, a replicon-cured cell line (BHK-F-Rep^cured^) with undetectable GFP expression was obtained after two consecutive treatments of 96 hours with 20 µM of GC376 (Fig. 2C). The BHK-F-Rep^cured^ cells served as a GFP-negative control (Fig. 2B) since the replicon-cured cell morphologically matched the BHK-F-Rep cell better than the parental BHK-21. In addition to GFP detection, immunofluorescence staining of the N-protein was performed as a control of the replication inhibition in an inhibitor experiment (Fig. 2D). Both the GFP expression and the expression of N diminished with higher concentrations of GC376, while the BHK-F-Rep^cured^ remained negative for both. As expected (Fig. 2E), the nucleocapsid immunofluorescence staining was slightly more sensitive than the GFP expression. A difference in the localization of the two signals was also noted. There seems to be an accumulation of N protein in the perinuclear regions, while GFP is distributed evenly throughout the cell. Collectively, these data demonstrate that the BHK-F-Rep cells provide a non-infectious experimental system to readily assess antiviral compounds using the median fluorescent intensity as a read-out for inhibition.

### Remdesivir and Nirmatrelvir, but not Amodiaquine are potent inhibitors of BHK-F-Rep

Next, we used the BHK-F-Rep cells to assess the inhibitory effects of Nirmatrelvir, Remdesivir, and Amodiaquine in comparison to GC376. Nirmatrelvir, like GC376, is a potent inhibitor of the coronavirus M^pro^, while Remdesivir is a ribonucleoside analog and inhibits viral RNA synthesis. The mode of action of Amodiaquine is not fully understood but it is suggested to act similarly to chloroquine through increasing endosomal pH and thus has antiviral activity against viruses that enter *via* endosomes. Although we expected that this inhibitory mechanism would not affect the replication of the replicon RNA in BHK-F-Rep cells, we included Amodiaquine because of previous reports of inhibitory effects on flaviviral replicon RNA cells (23).

**Fig 3:**
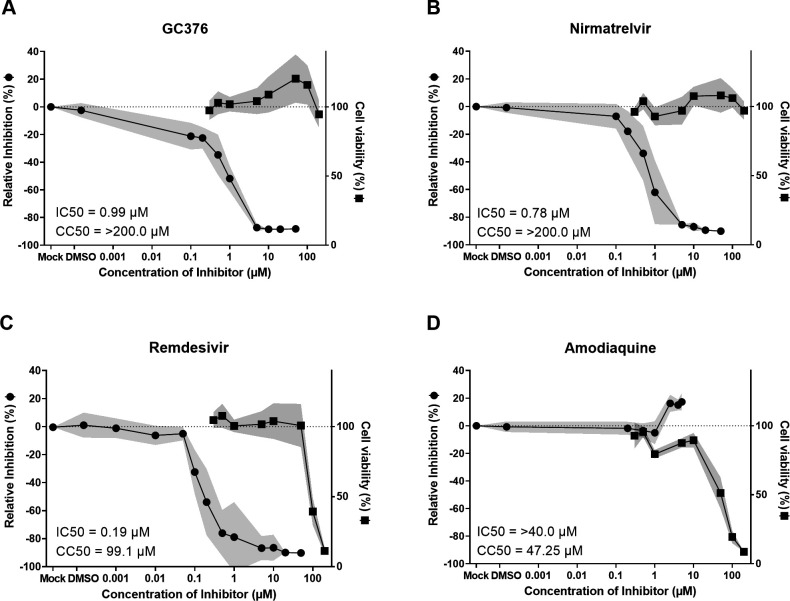
Individual tested antiviral compounds. BHK-F-Rep cells treated with (**A**) Remdesivir, (**B**) GC376, (**C**) Nirmatrelvir, and (**D**) Amodiaquine for 96 hours and analyzed by flow cytometry for GFP expression as shown in Fig. 2. Relative inhibition was calculated by the median intensity of GFP expression in the cells treated with antiviral drugs or DMSO in comparison to mock control. In addition, IC50 values and CC50 values were calculated as explained in the methods and represented on the individual graphs. (**A–C**) Remdesivir, GC376, and Nirmatrelvir showed effective antiviral dose-response with IC50 values < 1 µM, at concentrations lower than the cytotoxic concentrations (**D**) Amodiaquine alone did not show any inhibitory effect at the concentrations that already affected the cell growth The black line in the graphs represents the mean value of the data collected from three independent experiments, and the gray area represents standard error of the mean.

For the individually tested compounds, a clear dose-dependent inhibition of BHK-F-Rep cells could be detected with half-maximal inhibitory concentration (IC50s) values of 0.99 µM for GC376, 0.78 µM for Nirmatrelvir, and 0.19 µM for Remdesivir (Fig. 3; Table 1). Importantly, the 50% cytotoxic concentration (CC50) values of these compounds were 99.1 µM for Remdesivir and beyond the highest concentration used (200 µM) for GC376 and Nirmatrelvir (Fig. 3; Table 1). By contrast, we did not observe an inhibitory effect of Amodiaquine on BHK-F-Rep cells, suggesting that this compound does not target viral or host cell functions required for replication of the FECV replicon RNA.

**TABLE 1:** IC50, CC50, and SI values for antiviral compounds and combination of compounds on BHK-F-Rep cells

Compound	IC50 (µM)	CC50 (µM)	SI
Remdesivir	0.19	99.10	524.34
GC376	0.99	>200.00	>201.41
Nirmatrelvir	0.78	>200.00	>257.07
Amodiaquine	> 40.00	47.25	<1.18
0.2 µM Amodiaquine + GC376	1.78	>200.00	>112.36
0.5 µM Amodiaquine + GC376	1.80	>200.00	>111.11
0.2 µM Amodiaquine + Nirmatrelvir	1.00	>200.00	199.40
Remdesivir + GC376 (1:1)	0.15	99.10	656.29
Remdesivir + Nirmatrelvir (1:1)	0.17	99.10	596.99

### Assessment of inhibitor combinations in BHK-F-Rep cells and comparison to virus infection

**Fig 4:**
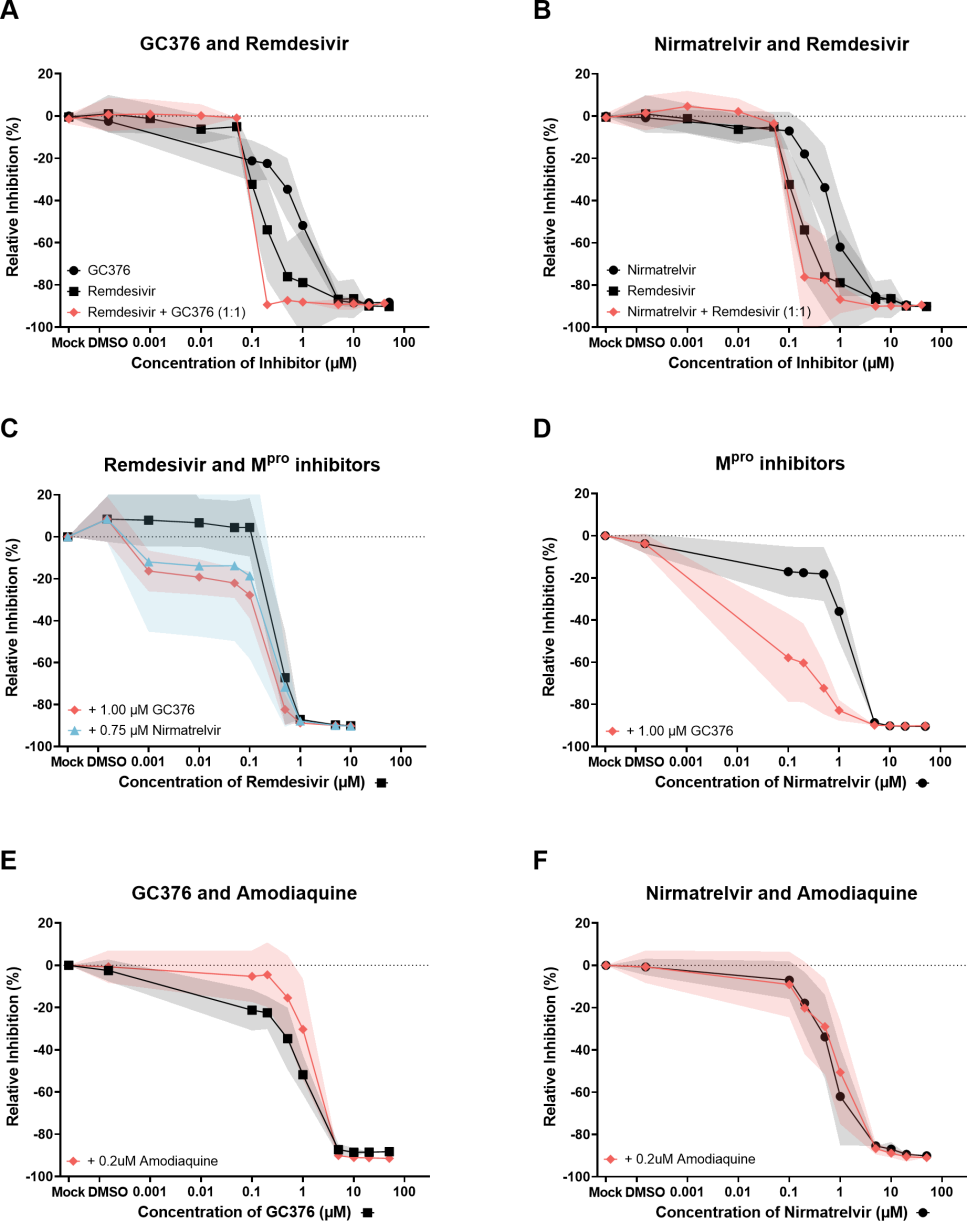
Combined antiviral compounds. BHK-F-Rep cells treated with different mixtures of Remdesivir, GC376, Nirmatrelvir, and Amodiaquine for 96 hours and analyzed by flow cytometry for GFP expression as shown in Fig. 2. (**A and B**) Combining Remdesivir in a 1:1 mixture with GC376 or Nirmatrelvir had a positive effect on IC50 values, making it more efficient in inhibiting replication of the FECV replicon. (**C**) *Combining either GC376 or Nirmatrelvir at their approximate IC50 values to Remdesivir to illustrate the additive effect of both M^pro^ inhibitors to Remdesivir.* (**D**) *Combining GC376 at its approximate IC50 value with different concentrations of Nirmatrelvir illustrates a highly increased additive effect when combining these two M^pro^ inhibitors.* (**E and F**) Adding Amodiaquine to either GC376 or Nirmatrelvir did not have a positive effect on the dose-response of either primary inhibitor. The black and red lines in the graphs represent the mean value of the data collected from three independent experiments of individual drugs or their combination, respectively. The gray and red shaded areas represent the standard error of the mean.

Considering the potential risk of developing drug resistance, especially in cases of relapse following treatment, it is important to develop drug combination therapies. Notably, combinations of compounds with different modes of action have, to our knowledge, not yet been tested for the treatment of FIP. However, Amodiaquine and the M^pro^ inhibitor GC376 have been assessed in combination *in vitro* with promising results. We therefore used BHK-F-Rep cells to assess different combinations of drugs. First, we evaluated compounds with different modes of action by combining the polymerase inhibitor Remdesivir with each of the two M^pro^ inhibitors GC376 or Nirmatrelvir, at a ratio of 1:1 with different dosages. As shown in Fig. 4A and B, we observed a dose-dependent inhibition when two inhibitors were combined that was comparable to the observed inhibition when the compounds were used individually. However, in the range of 0.1 to 1 µM where the inhibitors have their individual IC50s, there is a sharp increase in inhibition within a short range of escalating dosages, suggesting that the combination of two inhibitors with different viral targets may work additively. Next, we showed in Fig. 4C that when adding either GC376 or Nirmatrelvir, at their corresponding IC50 dosage to Remdesivir, the additive effect is more pronounced. Both M^pro^ inhibitors have an additive effect on Remdesivir. Furthermore, the combination of the two M^pro^ inhibitors GC376 and Nirmatrelvir was seen to be the most effective (Fig. 4D). In a last setting, we addressed the question of whether Amodiaquine may increase BHK-F-Rep inhibition in combination with the M^pro^ inhibitors GC376 or Nirmatrelvir. We added a constant amount of Amodiaquine (0.2 µM) to different dosages of GC376 or Nirmatrelvir. As shown in Fig. 4E and F, we did not observe any additive effect by adding Amodiaquine to the M^pro^ inhibitors, and in the case of the combination of Amodiaquine and GC376, inhibition appeared to be even negatively affected by Amodiaquine addition.

**Fig 5:**
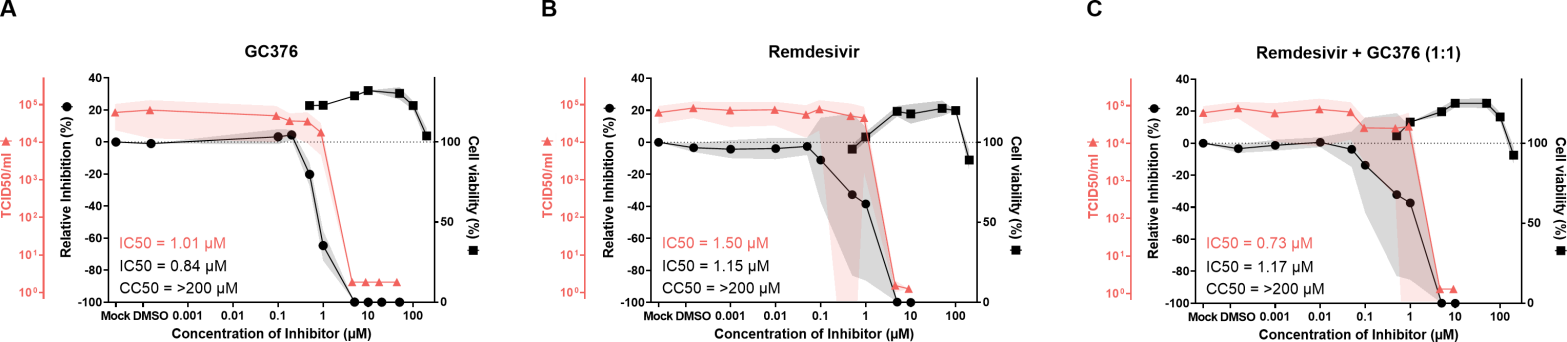
Comparison to virus infection. Testing of three different compounds or combinations of compounds: GC376 (**A**), Remdesivir (**B**), and Remdesivir + GC376 in a 1:1 ratio (**C**) in recFCoV-GFP-infected Fcwf-4 cells with dose-response curve of recFCoV-GFP infected Fcwf-4 cells treated for 48 hours. Read-out as with the BHK-F-Rep cells done *via* flow cytometry and detection of GFP-positive cells (relative Inhibition) including IC50 values are shown in black with the left y-axis. As with the BHK-F-Rep cells, data shown on cell viability testing in the recFCoV-GFP infected Fcwf-4 cells are shown in black with the right y-axis including CC50 values. Additional data on viral titers of the recFCoV-GFP infected Fcwf-4 cells treated with the inhibitors and corresponding IC50 values are shown in red. The black and red lines in the graphs represent the mean value of the data collected from three independent experiments of individual drugs or their combination, respectively. The gray and red shaded areas represent the standard error of the mean.

Finally, we assessed GC376 and Remdesivir individually or in combination in a virus infection system. We used the previously described recombinant recFCoV-GFP that is based on the laboratory-adapted serotype I FIPV strain Black and contains the GFP gene as a replacement for the accessory genes 3a–c (39). Using this virus, we could measure inhibition by assessing GFP expression or by virus titration. However, the assessment of GFP expression by measuring the median fluorescent intensity, as we did for BHK-F-rep cells, was not possible since recFCoV-GFP-infected *Felis catus* whole fetus 4 (Fcwf-4) cells died before the reduction of GFP expression was observable. Instead, we determined the number of GFP-positive cells to monitor inhibition (Fig. 5A through C). In parallel, we determined the virus titers released into the cell culture supernatant from inhibitor-treated and infected cells (Fig. 5A through C). To facilitate a direct comparison of the inhibitor assessment using infectious virus or the BHK-F-Rep cells, we chose a similar experimental setup by assessing the polymerase inhibitor Remdesivir and the M^pro^ inhibitor GC376 individually and in combination (ratio 1:1 at different dosages). The results are shown in Fig. 5C and can be compared with Fig. 4A (BHK-F-Rep inhibition) and IC50 values for the different experimental systems are shown in Table 2. The measurement of GFP expression (median fluorescent intensity for BHK-F-Rep cells and GFP-positive cells for virus-infected cells) revealed comparable dosages of inhibition for individual treatments with GC376 and Remdesivir in the range of 0.2 to 8 µM and with maximal inhibition starting at 10 µM. The combination of GC376 and Remdesivir however inhibits BHK-F-Rep cells already at 0.2 µM where inhibition assessed by measurement of GFP-positive and virus-infected cells is only partially detectable, suggesting that BHK-F-Rep cells are slightly more vulnerable to inhibition under these conditions. Notably, the IC50 values were generally lower for the treatment of BHK-F-Rep cells under conditions that included Remdesivir (Table 2). By measuring virus titers, inhibitory concentrations of individually, or in combination, applied GC376 and Remdesivir were generally higher but maximal inhibition is observed in the same range of 8–10 µM. Collectively, these data confirm that the BHK-F-Rep system is very well suited to assess the antiviral activities of several drugs that target viral functions required for RNA replication.

**TABLE 2:** IC50 values (µM) of Remdesivir, GC376, and combination of both in different experimental systems

	IC50 (µM)		
Compound	BHK-F-Rep flow cytometry	recFCoV-GFP flow cytometry	recFCoV-GFP viral titers
Remdesivir	0.19	1.15	1.50
GC376	0.99	0.84	1.01
Remdesivir + GC376 (1:1)	0.15	1.17	0.73

## DISCUSSION

Even though feline coronaviruses were discovered some time ago, it is currently still not possible to propagate serotype I feline coronaviruses on commercially available cell culture systems (40). Therefore, our approach of an FECV replicon system based on the genomic sequence of a serotype I FECV field strain poses an elegant and efficient system to study serotype I feline coronaviruses in more detail. Here, we show that the replicon is expressed and efficiently replicates in BHK cells. Furthermore, we also show that the FECV replicon system could be efficiently inhibited by multiple single as well as combined antiviral compounds. The efficiency of the FECV replicon system as an antiviral screening method was strongly supported by the comparable drug dose-response results of the infection of Fcwf-4 cells with the laboratory strain-adapted recFCoV-GFP. The antiviral compounds that successfully blocked the replicon system (Fig. 3 and 4) presented similar inhibition capacity in the *in vitro* infection model, detected using reduced GFP expression and diminished viral titers (Fig. 5).

Indrayanto et al. (41) proposed that a selectivity index (SI) of >10 should be considered to proceed with subsequent tests for future potential clinical use. For most of the tested substances and combinations of substances, except Amodiaquine, this was more than 10 times higher than the minimum SI required (Table 1). Therefore, all tested compounds and combinations could be of interest for future use, except Amodiaquine when used independently. As previously described, the addition of Amodiaquine to either GC376 or Nirmatrelvir had a negative influence on their individual effect; therefore, these should not be potential candidates either. The combinations of Remdesivir with GC376 or Remdesivir with Nirmatrelvir proved to be very efficient inhibitors and could potentially be of interest for future studies.

In addition, two of the most efficient inhibitors tested on the FECV replicon system, Remdesivir, and GC376, have already been shown by other research groups to be effective against FIPV *in vitro* as well as *in vivo* (13–20). Our screening results suggest that Nirmatrelvir would also be of interest for future *in vivo* studies, as it is already licensed for use in humans and could be easily applied in a veterinary setting. In addition, we propose that the use of a combination of antivirals would be a valuable approach, as there is a risk of cats developing resistance due to unlicensed and uncontrolled administration of compounds by cat owners. It is of great importance to have legally available treatment options against FIPV as soon as possible. Moreover, there have been reports that cats suffering from the neurological form of FIP are more difficult to treat (13, 14, 42, 43). It could be of interest to try out different combinations *in vivo* for these cats, to increase the likelihood of finding compounds crossing the blood-brain barrier. Therefore, having multiple treatment options already at hand would save many cats with different clinical presentations of FIP.

We expect that our FECV replicon system will be of great help to facilitate the identification and characterization of future anti-FIPV compounds. For example, anti-FIPV compounds with unknown target(s) or mode of action can be readily assessed if they affect RNA replication. Finally, the FECV replicon RNA will also be a valuable tool to study drug resistance mutations that may emerge in the future once antiviral FIP treatment has been widely established.

## MATERIALS AND METHODS

### Generation of the recombinant vaccinia virus containing the cDNA copy of the FECV replicon (vrecFECVrep)

The replicon used in this study is based on the genome sequence of an FECV field strain (strain Felix) described in Ehmann et al. (30). Genome positions refer to GenBank acc. no MG893511. The architecture of the replicon follows the structure of an already established human coronavirus 229E replicon (37). The replicon was generated by reverse genetics using an empty vaccinia virus vector based on the Western Reserve strain (vNotI/tk) (44). Four homologous recombination steps using *Escherichia coli* guanosine-phosphoribosyltransferase (GPT) as a selection marker were used to introduce the DNA copy of the FECV replicon into the vaccinia virus vector. The selection of recombinant vaccinia viruses was performed as described previously (45, 46). The recombinant vaccinia virus containing the FECV replicon cDNA sequence (vrecFECVrep) contains the following elements in this order: the bacteriophage T7 RNA polymerase promoter, FECV Felix genome position 1–632, the TaV 2A-like element from *Thosea asigna* virus (38), neomycin (*neo*) resistance gene derived from pTET/ON (Clontech), the EMCV IRES, an ATG start codon followed by FECV Felix genome position 632–20,416 (with an A20,413T silent mutation), an eGFP gene (mammalian codon-optimized), FECV Felix genome position 26,918–28,087 followed by FECV Felix genome position 28,773–29,298, a poly-A tail, a *ClaI* restriction site, and the sequence for the hepatitis delta ribozyme (HDR). The strategy for the generation of vrecFECVrep was based on four steps. The first round of recombination (pRE95) based on pGPT-1 (37) contains in the multi-cloning site (MCS) downstream of the GPT 500 bases from the vaccinia virus vector and the T7 promoter, FECV Felix 1–632, TaV 2A, *neo*, and FECV Felix 632–1,900. In the MCS upstream of GPT FECV Felix 19,900–20,416, GFP, FECV Felix 26,918–28,087 followed by FECV Felix 28,773–29,298, a poly A tail, the *ClaI* restriction site, and the HDR. The second plasmid used for recombination (pRE102) is based on pGemT and contains the genome sequence of FECV Felix positions 1,400–3,400 and 14,000–20,400. The third round of selection was done with plasmid pRE112. This plasmid, again, is based on pGPT-1 and contains the genome sequence of FECV Felix positions 3,000–5,500 and 9,500–14,500 in the MCS downstream and upstream of GPT. The plasmid for the final round of homologous recombination (pRE100) contains the genome sequence of FECV Felix positions 5,000–10,000. vrecFECVrep was then used to produce replicon RNA by *in vitro* transcription as described earlier (45).

### Generation of a plasmid expressing FECV nucleocapsid protein for transfection (pRE140)

To express homologous N protein in cells transfected with replicon RNA, a plasmid was generated containing the full-length sequence of the N gene of FECV Felix (genome positions 26,960–28,087). The N gene was amplified by PCR with primers adding a *NheI* and *NotI* restriction site and cloned into pCI-neo mammalian expression vector (Promega) using these enzymes. The *neo-*resistance cassette was subsequently deleted by a *KpnI* and *BamHI* restriction digestion, blunting with Klenow polymerase fragment (NEB) and subsequent religation with T4 DNA ligase (NEB) resulting in plasmid pRE140.

### Generation of a replicon cell line (BHK-F-Rep)

BHK cells were grown in Dulbecco’s modified Eagle’s medium (DMEM) supplemented with 10% fetal bovine serum (FBS), penicillin (100 U/mL), and streptomycin (0.1 mg/mL) in 5% CO_2_ at 37°C. For transfection, cells were seeded into six wells (500,000 cells per well), incubated overnight, and transfected with 10 µL of Lipofectamine 2000 (Invitrogen) and 2 µg of plasmid pRE140. Transfected cells were incubated for 24 hours. Then, cells from four wells were trypsinized, pooled, and used for electroporation with recFECVrep RNA as described earlier, generating the BHK cell line expressing recFECVrep (BHK-F-Rep) (45). As a negative control, the same number of cells was electroporated without replicon RNA. Electroporated cells were incubated for 48 hours to recover and proliferate. Then, cells were trypsinized and seeded in different concentrations (1:50; 1:100; 1:200) on 10 cm dishes to establish single-cell colonies. The medium was complemented with 600 µg G418/mL and changed daily. Cells were checked for green fluorescence each day. In all, 20 well-demarcated colonies with green fluorescence were picked, seeded in 24 wells, and propagated. The clone with the strongest fluorescence signal and healthy growth in cell culture was selected for further experiments and cryopreservation. The identity of the recFECVrep RNA was checked by RNA preparation and amplification of marker elements by RT-PCR.

### Cells and cell culture conditions

The BHK-F-Rep was maintained in Gibco Minimal Essential Medium with GlutaMAX (MEM; Thermo Fisher Scientific), supplemented with 10% fetal bovine serum (FBS; FBS Gold, PAN-Biotech) and 50 U/mL penicillin/streptomycin (Pen/Strep; Thermo Fisher Scientific or Seraglob). Selection pressure was maintained with 500 µg/mL of Geneticin (G418; Thermo Fisher Scientific). A cured cell line of the BHK-F-Rep was generated as a negative control (BHK-F-Rep^cured^) and was maintained also in MEM supplemented with 10% FBS and 50 U/mL Pen/Strep, without adding Geneticin as a selective agent. The BHK-F-Rep^cured^ was generated as follows: cells were passaged multiple times in T25 flasks and were then thinly seeded on a 96-well plate (100,000 cells/plate), and 20 µM of GC376 was added directly to the cells after splitting. The plate was incubated for 96 hours at 37°C and 5% CO_2_ and checked under the EVOS M700 imaging system. Multiple wells were selected where no GFP was detected and the same process with 20 µM of GC376 was repeated. The cells were then further passaged in T75 flasks until use. The *Felis catus* whole fetus 4 (Fcwf-4) cells were maintained in MEM, supplemented with 10% FBS, 50 U/mL Pen/Strep, and 1× Gibco MEM Non-Essential Amino Acids Solution (NEAA; Thermo Fisher Scientific).

### Viruses and bioinformatic analysis

The recombinant GFP-expressing cell-culture adapted FIPV (recFCoV-GFP) used in this study was generated and described earlier by Tekes et al. (39, 45). Sequence confirmation for both viruses was done by lysing cells (BHK-F-Rep or Fcwf-4 cells infected with the recFCoV-GFP) and extracting RNA with the NucleoSpin RNA Plus Kit (Macherey-Nagel) according to the manufacturer’s instructions. The RNA was quantified, and quality checked *via* NanoDrop Microvolume Spectrophotometer and sent for whole-genome NGS sequencing on the Illumina platform (NGS platform, University of Bern). Libraries for the vrecFECVrep sample were prepared using the TruSeq Stranded mRNA sample preparation kit.

For vrecFECVrep, sequencing reads were assessed for overall quality using FastQC v.0.11.9. Reads were trimmed and low-quality reads were removed *via* TrimGalore v.0.6.5. Trimmed reads were aligned to the vrecFECVrep reference sequence (created *in silico*) using Bowtie2 v.2.3.4. Samtools v.1.10 was used with the -d option set to 10,000 to generate a consensus sequence. Nucleotide variants were called using Lofreq v.2.1.5 with the -C option set to 100 and the -d option set to 10,000. The VCF file was filtered using the lofreq filter command for variants called at a frequency of 0.03. Data analysis was performed on UBELIX, the high-performance computing (HPC) cluster at the University of Bern (http://www.id.unibe.ch/hpc).

In addition, for the recFCoV-GFP a virus kinetics was performed, infecting Fcwf-4 cells with MOI of 0.1 and 0.01, incubating for 2 hours (MEM +1X NEAA +50 U/mL Pen/Strep), washing twice with phosphate-buffered saline (PBS; in-house) and taking samples every 24 hours for 7 days. Virus titration was performed with a TCID50 approach.

### RT-qPCR

To confirm the FECV replicon RNA transcription, primers and probes were designed to detect the EGFP and the RNA-dependent RNA polymerase (RdRp) gene. To confirm also the amount of subgenomic RNA, the forward primer was designed to bind in the 5′ untranslated region (UTR), the reverse primer was designed to bind either in the EGFP gene or the N gene and the probes were designed to bind on the converging site of the 5′ UTR and the EGFP gene or the 5′ UTR and the N gene (Table S1, Microsynth). For each tested condition, a cell pellet of 1 Mio cell was frozen at −20°C, and RNA was extracted with the Direct-zol RNA MiniPrep Kit (Zymo Research) as described by the manufacturer. RT-qPCR was performed with Applied Biosystems TaqMan Fast Virus 1-Step Master Mix (Thermo Fisher Scientific) as described by the manufacturer and was run on an Applied Biosystems PRISM 7500 Fast Sequence Detection System (Thermo Fisher Scientific) as described by the manufacturer. The RT-qPCRs were performed in duplicates including a negative control (nuclease-free-water, NFW).

### Inhibitors

Each tested inhibitor was diluted in dimethyl sulfoxide (DMSO) to the concentration used for each test condition. To each well 5 µL of inhibitor diluted in DMSO (1.10 g/mL) was added with 1 mL of medium (MEM +10% FBS +50 U/mL Pen/Strep). Therefore, for each condition, DMSO with a final concentration of 5.5 mg/mL was used, as well as the DMSO control for each tested condition. The following compounds were used: Remdesivir (MedChem Express), GC376 (Selleck Chemicals), Nirmatrelvir (PF-07321332; MedChem Express), and Amodiaquine dihydrochloride (Amodiaquine; MedChem Express).

### Inhibitor tests with the BHK-F-Rep

For each inhibitor test, the BHK-F-Rep was cultured in T75 or T150 flasks, washed once with PBS, cells detached with Gibco TrypLE Express (Trypsin; Thermo Fisher Scientific), and then seeded in 12-well plates (25,000 cells/well) in 1 mL/well of cell-culture medium (MEM +10% FBS +50 U/mL Pen/Strep). Directly after seeding, the inhibitor was added to the cells. The plates were incubated for 96 hours at 37°C with 5% CO_2_.

### Inhibitor tests with recFCoV-GFP

For each inhibitor test, the Fcwf-4 cells were cultured in T75 or T150 flasks, washed once with PBS, cells detached with trypsin, and then seeded in 12-well plates (250,000 cells/well) in 1 mL/well of cell-culture Medium (MEM +10% FBS +50 U/mL Pen/Strep + 1× NEAA). The plates were incubated for 24 hours at 37°C with 5% CO_2_. The medium was changed to infection medium (MEM +50 U/mL Pen/Strep + 1× NEAA) and then infected with the recFCoV-GFP at a multiplicity of infection (MOI) of 0.01. The cells were incubated for 2 hours at 37°C and 5% CO_2_ and then washed twice with PBS. 1 mL/well of new medium (MEM +10% FBS +50 U/mL Pen/Strep + 1× NEAA) was added and immediately afterward the inhibitor. The plates were then incubated for 48 hours at 37°C and 5% CO_2_. For the Fcwf-4 cells infected with the recFCoV-GFP cell, and the supernatant was taken from each tested condition and stored at −80°C for later titration.

### Immunofluorescence analysis

To perform indirect immunofluorescence analysis, the BHK-F-Rep cells were seeded in 8-Well Glass Bottom µ-Slides (ibid) and directly treated with the different concentrations of GC376 and kept at 37°C with 5% CO2 for 96 hours in their regular cell culture medium (MEM +10% FBS +50 U/mL Pen/Strep). After, the cells were washed twice with PBS and fixated at room temperature for 20 minutes with 4% of buffered formaldehyde solution (Formafix). After two washing steps with PBS, 0.1% Triton X-100 (in PBS) was added to each well and incubated for 5 min at room temperature. After removing this, CB buffer (PBS supplemented with 50 mM NH4Cl, 0.1% saponin, and 2% BSA) was added and incubated for 20 minutes at room temperature. Thereafter, the primary antibody solution [mouse anti-feline coronavirus FIPV3-70 (nucleocapsid)](Biorad) in a concentration of 1:200 was added and left to incubate for 2 hours at room temperature (protected from light). After three washing steps with CB buffer for 5 minutes each, the secondary antibody was added (donkey anti-mouse Alexa Fluor 647 (JIR) in a 1:400 dilution and kept at room temperature for 1 hour (protected from light). Again, the cells were washed three times afterward with CB buffer for 5 minutes each (protected from light), and then 2 drops of mounting medium [ProLong Diamond Antifade Mountant with DAPI (molecular probes)] were added per well. The slides were kept at 4°C, protected from light, until visualization with the DeltaVision Elite (General Electric) with a 40× (oil) or 100× (oil) objective and the DeltaVision Softworx Software. Image analysis was done with ImageJ 1.53t, all pictures per figure were analyzed with the same settings, and an overview was generated with the FigureJ Plugin of ImageJ 1.53t.

### Flow cytometry

To assess the GFP expression in BHK and Fcwf-4 cells, the supernatant was removed, the cells were washed once with PBS and then detached with trypsin and transferred to a 96-well plate. The plate was centrifuged at 1,700 rpm for 5 minutes and the supernatant discarded. Cells were once washed again with PBS and again centrifuged at 1,700 rpm for 5 minutes and the supernatant was discarded. For the replicon conditions, the cells were then stained with the LIVE/DEAD Fixable Aqua Dead Cell Stain Kit for 405 nm excitation (Invitrogen) according to the manufacturer’s instructions. As the dead cell control, one of the samples was placed at 60°C for 20 minutes and then stained. After staining, the cells were washed with PBS and centrifuged (1,700 rpm for 5 minutes), and the supernatant was discarded. For the Fcwf-4 cells infected with the recFCoV-GFP, the staining and washing step was not performed. Then, all the cells were washed with PBS before being resuspended in 200 µL PBS and transferred to Falcon 5 mL round-bottom tubes with cell strainer cap (Corning Incorporated) and kept in the dark on ice. Samples were acquired in Cytek Aurora with SpectroFlo Software Version 3.1.0 and analyzed using FlowJo software (FlowJo_v108.1).

For the BHK-F-Rep, median fluorescence intensity was taken as a readout, since the number of GFP-positive (GFP^+^) cells fluctuated between different passages and median intensity remained more consistent between passages. However, this was not possible for the recFCoV-GFP-infected Fcwf-4 cells since they died before the reduction of GFP expression could be observed. Instead, to monitor inhibition, we determined the percent of GFP-positive cells. In either case, the median intensity of the GFP signal or the percentage of GFP-positive cells was normalized to the measurements of untreated conditions, by defining the untreated results of each triplicate as 100%. The value was then subtracted by 100 to get the value representing the percentage of inhibition.

### Read-out *via* TCID50

As described previously for each condition, the supernatant was harvested after the incubation period of 48 hours at 37°C and 5% CO_2_ and frozen at −80°C. 50% tissue culture infectious doses assay (TCID50) was performed on Fcwf-4 cells in six replicates on 96-well plates. The Fcwf-4 cells were seeded at 250,000 cells/plate and kept for 24 hours at 37°C and 5% CO_2_. Then, a 1:10 dilution Series of the virus sample was performed, and the cells were kept for another 96 hours at 37°C and 5% CO_2_. Samples were then fixed and inactivated with 4% buffered formaldehyde solution (Formafix) and stained with crystal violet (Sigma-Aldrich). TCID50 calculations were performed with the Spearman Kärber method (47).

### *In vitro* cytotoxicity assay

To define cytotoxic concentrations of the individual inhibitors, cytotoxicity assay with the CytoTox-Glo Cytotoxicity Assay (Promega) was performed according to the manufacturer’s instructions. As a positive control, 75 µg/well of cycloheximide was used. The inhibitors were diluted in DMSO to concentrations so that 2 µL/well could be added to reach the tested concentrations. For luminescence detection, a Hidex Sense with the HidexPlateReader Software 1.0.0 was used. Detection parameters were set for luminescence detection with an infrared cutoff of 1 second. As described by Promega, the difference of viable vs dead cells was calculated in Microsoft Excel 2016, and 50% of cytotoxic concentration (CC50) was determined in GraphPad Prism version 9.3.1 for Windows. The data were fitted to a nonlinear regression curve with log(inhibitor) vs normalized response with variable slope.

### Graphs and statistical analysis

All conditions were tested in triplicates on three different days always including a negative control. Statistical analysis and generation of graphs were performed using GraphPad Prism version 9.3.1 for Windows. The same as CC50, 50% inhibitory concentration (IC50) was determined in GraphPad Prism version 9.3.1 for Windows. The Data were fitted to a nonlinear regression curve with log(inhibitor) vs normalized response with variable slope. The selectivity index (SI) was calculated in Microsoft Excel 2016 as follows: SI = CC50/IC50.

## SUPPLEMENTAL MATERIAL

Table S1 jvi.01216-23-s0001.docx vrecFECVrep polymorphisms with allele frequency >20%.

Table S2 jvi.01216-23-s0002.docx Primer list for RT-qPCR.

ASM does not own the copyrights to Supplemental Material that may be linked to, or accessed through, an article. The authors have granted ASM a non-exclusive, world-wide license to publish the Supplemental Material files. Please contact the corresponding author directly for reuse.

## References

[1] Thayer V, Gogolski S, Felten S, Hartmann K, Kennedy M, Olah GA. 2022. 2022 AAFP/EveryCat feline infectious peritonitis diagnosis guidelines. J Feline Med Surg 24:905–933. doi:10.1177/1098612X221118761 36002137 PMC10812230

[2] Pedersen NC. 2014. An update on feline infectious peritonitis: virology and immunopathogenesis. Vet J 201:123–132. doi:10.1016/j.tvjl.2014.04.017 24837550 PMC7110662

[3] Kennedy MA. 2020. Feline infectious peritonitis: update on pathogenesis, diagnostics, and treatment. Vet Clin North Am Small Anim Pract 50:1001–1011. doi:10.1016/j.cvsm.2020.05.002 32563530

[4] Pedersen NC. 2009. A review of feline infectious peritonitis virus infection: 1963-2008. J Feline Med Surg 11:225–258. doi:10.1016/j.jfms.2008.09.008 19254859 PMC7129802

[5] de Groot RJ, Baker SC, Baric R, Enjuanes L, Gorbalenya AE, Holmes KV, Perlman S, Poon L, Rottier PJM, Talbot PJ. 2011. Family Coronaviridae

[6] Addie D, Belák S, Boucraut-Baralon C, Egberink H, Frymus T, Gruffydd-Jones T, Hartmann K, Hosie MJ, Lloret A, Lutz H, Marsilio F, Pennisi MG, Radford AD, Thiry E, Truyen U, Horzinek MC. 2009. Feline infectious peritonitis. ABCD guidelines on prevention and management. J Feline Med Surg 11:594–604. doi:10.1016/j.jfms.2009.05.008 19481039 PMC7129471

[7] Desmarets LMB, Vermeulen BL, Theuns S, Conceição-Neto N, Zeller M, Roukaerts IDM, Acar DD, Olyslaegers DAJ, Van Ranst M, Matthijnssens J, Nauwynck HJ. 2016. Experimental feline enteric coronavirus infection reveals an aberrant infection pattern and shedding of mutants with impaired infectivity in enterocyte cultures. Sci Rep 6:20022. doi:10.1038/srep20022 26822958 PMC4731813

[8] Kipar A, Meli ML, Baptiste KE, Bowker LJ, Lutz H. 2010. Sites of feline coronavirus persistence in healthy cats. J Gen Virol 91:1698–1707. doi:10.1099/vir.0.020214-0 20237226

[9] Pedersen NC, Allen CE, Lyons LA. 2008. Pathogenesis of feline enteric coronavirus infection. J Feline Med Surg 10:529–541. doi:10.1016/j.jfms.2008.02.006 18538604 PMC7130060

[10] Vogel L, Van der Lubben M, te Lintelo EG, Bekker CPJ, Geerts T, Schuijff LS, Grinwis GCM, Egberink HF, Rottier PJM. 2010. Pathogenic characteristics of persistent feline enteric coronavirus infection in cats. Vet Res 41:71. doi:10.1051/vetres/2010043 20663472 PMC2939696

[11] Pedersen NC, Liu H, Scarlett J, Leutenegger CM, Golovko L, Kennedy H, Kamal FM. 2012. Feline infectious peritonitis: role of the feline coronavirus 3C gene in intestinal tropism and pathogenicity based upon isolates from resident and adopted shelter cats. Virus Res 165:17–28. doi:10.1016/j.virusres.2011.12.020 22280883 PMC7114484

[12] Delaplace M, Huet H, Gambino A, Le Poder S. 2021. Feline coronavirus antivirals: a review. Pathogens 10:1150. doi:10.3390/pathogens10091150 34578182 PMC8469112

[13] Murphy BG, Perron M, Murakami E, Bauer K, Park Y, Eckstrand C, Liepnieks M, Pedersen NC. 2018. The nucleoside analog GS-441524 strongly inhibits feline infectious peritonitis (FIP) virus in tissue culture and experimental cat infection studies. Vet Microbiol 219:226–233. doi:10.1016/j.vetmic.2018.04.026 29778200 PMC7117434

[14] Pedersen NC, Perron M, Bannasch M, Montgomery E, Murakami E, Liepnieks M, Liu H. 2019. Efficacy and safety of the nucleoside analog GS-441524 for treatment of cats with naturally occurring feline infectious peritonitis. J Feline Med Surg 21:271–281. doi:10.1177/1098612X19825701 30755068 PMC6435921

[15] Dickinson PJ, Bannasch M, Thomasy SM, Murthy VD, Vernau KM, Liepnieks M, Montgomery E, Knickelbein KE, Murphy B, Pedersen NC. 2020. Antiviral treatment using the adenosine nucleoside analogue GS-441524 in cats with clinically diagnosed neurological feline infectious peritonitis. J Vet Intern Med 34:1587–1593. doi:10.1111/jvim.15780 32441826 PMC7379040

[16] Yin Y, Li T, Wang C, Liu X, Ouyang H, Ji W, Liu J, Liao X, Li J, Hu C. 2021. A retrospective study of clinical and laboratory features and treatment on cats highly suspected of feline infectious peritonitis in Wuhan, China. Sci Rep 11:5208. doi:10.1038/s41598-021-84754-0 33664426 PMC7970852

[17] Addie DD, Covell-Ritchie J, Jarrett O, Fosbery M. 2020. Rapid resolution of non-effusive feline infectious peritonitis uveitis with an oral adenosine nucleoside analogue and feline interferon omega. Viruses 12:1216. doi:10.3390/v12111216 33121021 PMC7693373

[18] Perera KD, Rathnayake AD, Liu H, Pedersen NC, Groutas WC, Chang K-O, Kim Y. 2019. Characterization of amino acid substitutions in feline coronavirus 3C-like protease from a cat with feline infectious peritonitis treated with a protease inhibitor. Vet Microbiol 237:108398. doi:10.1016/j.vetmic.2019.108398 31585653 PMC6779346

[19] Kim Y, Lovell S, Tiew K-C, Mandadapu SR, Alliston KR, Battaile KP, Groutas WC, Chang K-O. 2012. Broad-spectrum antivirals against 3C or 3C-like proteases of picornaviruses, noroviruses, and coronaviruses. J Virol 86:11754–11762. doi:10.1128/JVI.01348-12 22915796 PMC3486288

[20] Kim Y, Shivanna V, Narayanan S, Prior AM, Weerasekara S, Hua DH, Kankanamalage ACG, Groutas WC, Chang K-O, Perlman S. 2015. Broad-spectrum inhibitors against 3C-like proteases of feline coronaviruses and feline caliciviruses. J Virol 89:4942–4950. doi:10.1128/JVI.03688-14 25694593 PMC4403489

[21] D’Alessandro S, Scaccabarozzi D, Signorini L, Perego F, Ilboudo DP, Ferrante P, Delbue S. 2020. The use of antimalarial drugs against viral infection. Microorganisms 8:85. doi:10.3390/microorganisms8010085 31936284 PMC7022795

[22] Al-Bari M. 2017. Targeting endosomal acidification by chloroquine analogs as a promising strategy for the treatment of emerging viral diseases. Pharmacol Res Perspect 5:e00293. doi:10.1002/prp2.293 28596841 PMC5461643

[23] Boonyasuppayakorn S, Reichert ED, Manzano M, Nagarajan K, Padmanabhan R. 2014. Amodiaquine, an antimalarial drug, inhibits dengue virus type 2 replication and infectivity. Antiviral Res 106:125–134. doi:10.1016/j.antiviral.2014.03.014 24680954 PMC4523242

[24] Mackenzie AH. 1983. Pharmacologic actions of 4-aminoquinoline compounds. Am J Med 75:5–10. doi:10.1016/0002-9343(83)91264-0 6603166

[25] Cook SE, Vogel H, Castillo D, Olsen M, Pedersen N, Murphy BG. 2022. Investigation of monotherapy and combined anticoronaviral therapies against feline coronavirus serotype II in vitro. J Feline Med Surg 24:943–953. doi:10.1177/1098612X211048647 34676775 PMC10812298

[26] Vangeel L, Chiu W, De Jonghe S, Maes P, Slechten B, Raymenants J, André E, Leyssen P, Neyts J, Jochmans D. 2022. Remdesivir, molnupiravir and Nirmatrelvir remain active against SARS-CoV-2 omicron and other variants of concern. Antiviral Res 198:105252. doi:10.1016/j.antiviral.2022.105252 35085683 PMC8785409

[27] Owen DR, Allerton CMN, Anderson AS, Aschenbrenner L, Avery M, Berritt S, Boras B, Cardin RD, Carlo A, Coffman KJ, et al.. 2021. An oral SARS-CoV-2 M^pro^ inhibitor clinical candidate for the treatment of COVID-19. Science 374:1586–1593. doi:10.1126/science.abl4784 34726479

[28] Ullrich S, Ekanayake KB, Otting G, Nitsche C. 2022. Main protease mutants of SARS-CoV-2 variants remain susceptible to Nirmatrelvir. Bioorg Med Chem Lett 62:128629. doi:10.1016/j.bmcl.2022.128629 35182772 PMC8856729

[29] Carlin AF, Beadle JR, Clark AE, Gully KL, Moreira FR, Baric RS, Graham RL, Valiaeva N, Leibel SL, Bray W, McMillan RE, Freshman JE, Garretson AF, McVicar RN, Rana T, Zhang X-Q, Murphy JA, Schooley RT, Hostetler KY. 2023. 1- O-octadecyl-2- O-benzyl- sn-glyceryl-3- phospho-GS-441524 (V2043). Evaluation of oral V2043 in a mouse model of SARS-CoV-2 infection and synthesis and antiviral evaluation of additional phospholipid esters with enhanced anti-SARS-CoV-2 activity. J Med Chem 66:5802–5819. doi:10.1021/acs.jmedchem.3c00046 37040439 PMC10108740

[30] Ehmann R, Kristen-Burmann C, Bank-Wolf B, König M, Herden C, Hain T, Thiel HJ, Ziebuhr J, Tekes G. 2018. Reverse genetics for type I feline coronavirus field isolate to study the molecular pathogenesis of feline infectious peritonitis. mBio 9:e01422-18. doi:10.1128/mBio.01422-18 30065095 PMC6069117

[31] Lohmann V, Körner F, Koch J-O, Herian U, Theilmann L, Bartenschlager R. 1999. Replication of subgenomic hepatitis C virus RNAs in a hepatoma cell line. Science 285:110–113. doi:10.1126/science.285.5424.110 10390360

[32] Bartenschlager R. 2005. The hepatitis C virus replicon system: from basic research to clinical application. J Hepatol 43:210–216. doi:10.1016/j.jhep.2005.05.013 15964655

[33] Westaway EG, Mackenzie JM, Khromykh AA. 2003. Kunjin RNA replication and applications of Kunjin replicons. Adv Virus Res 59:99–140. doi:10.1016/s0065-3527(03)59004-2 14696328

[34] Thiel V, Siddell SG. 2005. Reverse genetics of coronaviruses using vaccinia virus vectors, p 199–227. In Enjuanes L (ed), Coronavirus replication and reverse genetics. Springer, Berlin Heidelberg. 10.1007/3-540-26765-4_7 PMC8387696 15609513

[35] Ricardo-Lax I, Luna JM, Thao TTN, Le Pen J, Yu Y, Hoffmann H-H, Schneider WM, Razooky BS, Fernandez-Martinez J, Schmidt F, et al.. 2021. Replication and single-cycle delivery of SARS-CoV-2 replicons. Science 374:1099–1106. doi:10.1126/science.abj8430 34648371 PMC9007107

[36] Thiel V, Herold J, Schelle B, Siddell SG. 2001. Viral replicase gene products suffice for coronavirus discontinuous transcription. J Virol 75:6676–6681. doi:10.1128/JVI.75.14.6676-6681.2001 11413334 PMC114390

[37] Hertzig T, Scandella E, Schelle B, Ziebuhr J, Siddell SG, Ludewig B, Thiel V. 2004. Rapid identification of coronavirus replicase inhibitors using a selectable replicon RNA. J Gen Virol 85:1717–1725. doi:10.1099/vir.0.80044-0 15166457

[38] Donnelly MLL, Hughes LE, Luke G, Mendoza H, Ten Dam E, Gani D, Ryan MD. 2001. The ‘cleavage’ activities of foot-and-mouth disease virus 2A site-directed mutants and naturally occurring ‘2A-like’ sequences. J Gen Virol 82:1027–1041. doi:10.1099/0022-1317-82-5-1027 11297677

[39] Tekes G, Hofmann-Lehmann R, Bank-Wolf B, Maier R, Thiel H-J, Thiel V. 2010. Chimeric feline coronaviruses that encode type II spike protein on type I genetic background display accelerated viral growth and altered receptor usage. J Virol 84:1326–1333. doi:10.1128/JVI.01568-09 19906918 PMC2812337

[40] Desmarets LMB, Theuns S, Olyslaegers DAJ, Dedeurwaerder A, Vermeulen BL, Roukaerts IDM, Nauwynck HJ. 2013. Establishment of feline intestinal epithelial cell cultures for the propagation and study of feline enteric coronaviruses. Vet Res 44:71. doi:10.1186/1297-9716-44-71 23964891 PMC3765525

[41] Indrayanto G, Putra GS, Suhud F. 2021. Validation of in-vitro bioassay methods: application in herbal drug research. Profiles Drug Subst Excip Relat Methodol 46:273–307. doi:10.1016/bs.podrm.2020.07.005 33461699

[42] Kim Y, Liu H, Galasiti Kankanamalage AC, Weerasekara S, Hua DH, Groutas WC, Chang K-O, Pedersen NC, Perlman S. 2016. Reversal of the progression of fatal coronavirus infection in cats by a broad-spectrum coronavirus protease inhibitor. PLoS Pathog 12:e1005531. doi:10.1371/journal.ppat.1005531 27027316 PMC4814111

[43] Pedersen NC, Kim Y, Liu H, Galasiti Kankanamalage AC, Eckstrand C, Groutas WC, Bannasch M, Meadows JM, Chang KO. 2018. Efficacy of a 3C-like protease inhibitor in treating various forms of acquired feline infectious peritonitis. J Feline Med Surg 20:378–392. doi:10.1177/1098612X17729626 28901812 PMC5871025

[44] Merchlinsky M, Moss B. 1992. Introduction of foreign DNA into the vaccinia virus genome by in vitro ligation: recombination-independent selectable cloning vectors. Virology 190:522–526. doi:10.1016/0042-6822(92)91246-q 1529553

[45] Tekes G, Hofmann-Lehmann R, Stallkamp I, Thiel V, Thiel H-J. 2008. Genome organization and reverse genetic analysis of a type I feline coronavirus. J Virol 82:1851–1859. doi:10.1128/JVI.02339-07 18077720 PMC2258703

[46] Thiel V, Herold J, Schelle B, Siddell SG. 2001. Infectious RNA transcribed in vitro from a cDNA copy of the human coronavirus genome cloned in vaccinia virus. J Gen Virol 82:1273–1281. doi:10.1099/0022-1317-82-6-1273 11369870

[47] Hierholzer JC, Killington RA. 1996. 2 - virus isolation and quantitation, p 25–46. In Mahy BWJ, Kangro HO (ed), Virology methods manual. Academic Press, London.

