# Supplementary material for: An RNA replicon system to investigate promising inhibitors of feline coronavirus
Source: J Virol. 2024 Jan 18;98(2):e01216-23. doi: 10.1128/jvi.01216-23 (PMC10878086; doi:10.1128/jvi.01216-23)
Supplement: Table S1 — vrecFECVrep polymorphisms with allele frequency >20%. [file jvi.01216-23-s0001.docx]

**Supplementary data**

**Sequence analysis**

**Supplementary Table 1:** vrecFECVrep polymorphisms with allele frequency >20%. AA = Amino Acid.

| **Position** | **Mutation** | **AA Change** | **Gene** | **Allele Frequency (%)** |
| --- | --- | --- | --- | --- |
| 332 | G 🡪 T | K 🡪 N | nsp 1 | 99.75 |
| 666 | T 🡪 C | C 🡪 R | T2A | 33.88 |
| 1518 | A 🡪 G |  | IRES | 98.04 |
| 1634 | C 🡪 A |  | IRES | 56.35 |
| 1936 | A 🡪 T |  | IRES | 99.52 |
| 12946 | T 🡪 A | silent | nsp 8 | 99.92 |
| 13438 | T 🡪 C | silent | nsp 10 | 99.78 |
| 15492 | G 🡪 A | R 🡪 Q | nsp 12 | 99.78 |
| 16366 | C 🡪 T | silent | nsp 12 | 99.90 |
| 17049 | T 🡪 C | silent | nsp 13 | 99.95 |
| 17105 | C 🡪 T | S 🡪 F | nsp 13 | 98.13 |
| 17283 | G 🡪 A | silent | nsp 13 | 99.77 |
| 18696 | T 🡪 A | silent | nsp 14 | 98.20 |
| 19715 | A 🡪 T | D 🡪 V | nsp 14 | 98.42 |
| 21477 | C 🡪 T | silent | nsp 16 | 98.34 |
