# Supplementary material for: An RNA replicon system to investigate promising inhibitors of feline coronavirus
Source: J Virol. 2024 Jan 18;98(2):e01216-23. doi: 10.1128/jvi.01216-23 (PMC10878086; doi:10.1128/jvi.01216-23)
Supplement: Table S2 — Primer list for RT-qPCR. [file jvi.01216-23-s0002.docx]

**Supplementary Table 2:** Primer list for the RT-qPCR of the RdRp, EGFP, subgenomic GFP and subgenomic N gene. nt = nucleotides, TM = Melting Temperature in °C.

| **Name** | **Gene** | **Sequence** | **nt** | **TM** | **Reporter** | **Quencher** |
| --- | --- | --- | --- | --- | --- | --- |
| Genomic EGFP |  |  |  |  |  |  |
| EGFP2_frw | EGFP | GGGCACAAGCTGGAGTACAAC | 21 | 58.3 | na | na |
| EGFP2_rev | EGFP | CACCTTGATGCCGTTCTTCTG | 21 | 59.3 | na | na |
| EGFP2_pro | EGFP | ACAACAGCCACAACGTCTATATCATGGCC | 29 | 68.1 | Cy5 | BHQ2 |
| Genomic RdRp |  |  |  |  |  |  |
| FCoV_RdRp_F16 | RdRp | AGCGTTGTACTAAGAGCGTTATGGA | 25 | 59.4 | na | na |
| FCoV_RdRp_R17 | RdRp | CACATCGACCTTCCTTATACAAAAAG | 26 | 58.3 | na | na |
| FCoV_RdRp_mgb18 | RdRp | ATGAGCAAGTCTGTTATAAC | 20 | 70.0 | Fam | MQ530 |
| Subgenomic EGFP |  |  |  |  |  |  |
| FCoV_5utr_F19 | 5utr | TTAACTAGCCTTGTGCTAGATTTGTCTTC | 29 | 59.8 | na | na |
| FCoV_eGFP_R20 | EGFP | GCACCACCCCGGTGAAC | 17 | 59.3 | na | na |
| FCoV_5utr-eGFP_mgb21 | 5utr/EGFP | ACTCGAACTAAACTTTGGTAAT | 22 | 69.0 | Fam | MQ530 |
| Subgenomic N |  |  |  |  |  |  |
| FCoV_5utr_F19 | 5utr | TTAACTAGCCTTGTGCTAGATTTGTCTTC | 29 | 59.8 | na | na |
| FCoV_N_R22 | N | ATTCTTCCGACCACGAGAGTTAGA | 24 | 59.3 | na | na |
| FCoV_5utr-N_mgb23 | 5utr/N | CAACTCGAACTAAACTTCTAAAT | 23 | 70.0 | Fam | MQ530 |
